# Temporal Expression of Apelin/Apelin Receptor in Ischemic Stroke and its Therapeutic Potential

**DOI:** 10.3389/fnmol.2017.00001

**Published:** 2017-01-23

**Authors:** Yili Wu, Xin Wang, Xuan Zhou, Baohua Cheng, Gongying Li, Bo Bai

**Affiliations:** ^1^Department of Psychiatry, Jining Medical UniversityJining, China; ^2^Collaborative Innovation Center for Birth Defect Research and Transformation of Shandong Province, Jining Medical UniversityJining, China; ^3^Shandong Key Laboratory of Behavioral Medicine, Jining Medical UniversityJining, China; ^4^Neurobiology Institute, Jining Medical UniversityJining, China

**Keywords:** apelin, apelin receptor (APLNR), ischemic stroke, temporal expression, neuroprotection

## Abstract

Stroke is one of the leading causes of death and disability worldwide, and ischemic stroke accounts for approximately 87% of cases. Improving post-stroke recovery is a major challenge in stroke treatment. Accumulated evidence indicates that the apelinergic system, consisting of apelin and apelin receptor (APLNR), is temporally dysregulated in ischemic stroke. Moreover, the apelinergic system plays a pivotal role in post-stroke recovery by inhibiting neuronal apoptosis and facilitating angiogenesis through various molecular pathways. In this review article, we summarize the temporal expression of apelin and APLNR in ischemic stroke and the mechanisms of their dysregulation. In addition, the protective role of the apelinergic system in ischemic stroke and the underlying mechanisms of its protective effects are discussed. Furthermore, critical issues in activating the apelinergic system as a potential therapy will also be discussed. The aim of this review article is to shed light on exploiting the activation of the apelinergic system to treat ischemic stroke.

## Introduction

Stroke is one of the leading causes of death and disability worldwide and the majority of cases are ischemic stroke. To improve recovery and reduce disability, novel therapeutic approaches need to be developed, as current treatments are only limited to thrombolytic therapy within an extremely narrow time window (Craig and Housley, 2016). A growing body of evidence indicates that the apelinergic system, consisting of apelin and apelin receptor (APLNR), is implicated in ischemic stroke. First, Hata et al. (2007, 2011) showed that a single-nucleotide polymorphism (SNP, rs9943582) in APLNR is significantly associated with the increased risk of ischemic stroke in human. Moreover, the expression of apelin and APLNR is temporally dysregulated during different phases of ischemic stroke. Importantly, a number of studies showed that the apelinergic system plays a pivotal role in protecting cells from ischemic stroke-induced apoptosis and improving behavioral performance. The aforementioned evidence indicates that the apelinergic system might be a potential target for novel treatments. Therefore, we summarize the temporal expression of apelin and APLNR in ischemic stroke, the mechanisms of their dysregulation, the protective effect of the apelinergic system on ischemic stroke and the underlying mechanisms of its protective effect. In addition, we also discuss critical issues that may affect strategies for activating the apelinergic system as a potential therapy.

## Overview of Ischemic Stroke

### Epidemiology and Prevalence of Ischemic Stroke

Stroke, caused by the disturbance of blood supply to the brain, is the second leading cause of death and the third leading cause of disability worldwide (Wang et al., 2012). In the United States, stroke is the leading cause of long-term disability, including both physical and cognitive deficits. By 2030, the prevalence of stroke will increase by more than 20% over 2012 and the direct medical costs are projected to reach $184.1 billion, which is a great challenge to human society and healthcare system (Mozaffarian et al., 2016). The situation is much worse in China, where stroke is the leading cause of death (Wang H. et al., 2016). Ischemic stroke is caused by the blockage of an artery in the brain, accounting for approximately 87% of stroke cases, which contributes to the major portion of death and post-stroke disability in patients (Mozaffarian et al., 2016). In addition to acute brain damage, stroke significantly increases the risk of neurodegenerative diseases, such as Alzheimer’s disease (Wu et al., 2014). However, current treatments are only limited to thrombolytic therapy within an extremely narrow time window. Therefore, it is urgent to develop novel therapeutic approaches for stroke treatment.

### Mechanisms of Cell Death in Ischemic Stroke

Depending on the severity of lack of blood supply, the ischemic territory consists of the ischemic core and penumbra, which are respectively characterized by acute and delayed cell death, necrosis and apoptosis (Lipton, 1999; Lo, 2008). Ischemic core, the center area of the ischemic territory, is caused by an acute blood reduction to less than 15%, while ischemic penumbra, the surrounding area of the core, has a higher blood supply. Necrosis occurs within minutes after stroke, thus, cells within the ischemic core cannot be rescued. However, cells in the penumbra experience less severe blood reduction and may undergo apoptosis (Lipton, 1999; Lo, 2008). Although cells in the penumbra are vulnerable, their death is salvageable by proper intervention, suggesting that preventing penumbra cell death is the key to improve the recovery from and the prognosis of ischemic stroke. During acute ischemia, the diffusion of toxic contents released from dead cells in the core region triggers apoptotic responses. In addition, ischemia/reperfusion injury contributes to the apoptosis of penumbral cells by a number of molecular mechanisms, such as oxidative stress, inflammatory response, endoplasmic reticulum (ER) stress and abnormal protein degradation (Moskowitz et al., 2010; Zhang et al., 2014b). Therefore, blocking the detrimental pathways has a therapeutic potential to reduce penumbra cell death and improve stroke recovery.

## The Apelinergic System—Apelin and APLNR

### Apelin

The apelin (APLN) gene, located on chromosome Xq25, spans 9698bp, consisting of three exons and two introns. It encodes the preproapelin of 77 amino acids. Various mature peptides are derived from the 55 amino-acid propeptide, ranging from 13–36 amino acids. The mature peptides are highly conserved between species. In particular, the C-terminal 23 amino acids are 100% *homologous* between human and rat, mouse or bovine. Tatemoto et al. (1998) first identified apelin-36 *in vivo* and predicted it can be further processed to apelin-17 and apelin-13 with 17 and 13 amino acids, respectively. In addition, the shorter C-terminal peptides, apelin-13 and its pyroglutamate-modified form Pyr-apelin-13, have higher activity compared with apelin-36 (Hosoya et al., 2000). Mesmin et al. (2011) identified 46 endogenous apelin peptides in bovine colostrums. Apelin-13, the most active isoform, has been widely studied in ischemia/reperfusion injury (Tao et al., 2011; Khaksari et al., 2012; Yang et al., 2014, 2015; Chen et al., 2015; Li et al., 2016). Although all apelin isoforms may function through the unique APLNR, their tissue specificity, binding affinity to APLNR and efficacy in APLNR recycling may lead to differential functions of isoforms. However, the proapelin processing and the proteases involved in the generation of apelin isoforms have received less investigation. Recently, Shin et al. (2013) showed that proprotein convertase subtilisin/kexin 3 (PCSK3) directly cleaves proapelin to generate apelin-13.

**Figure 1:**
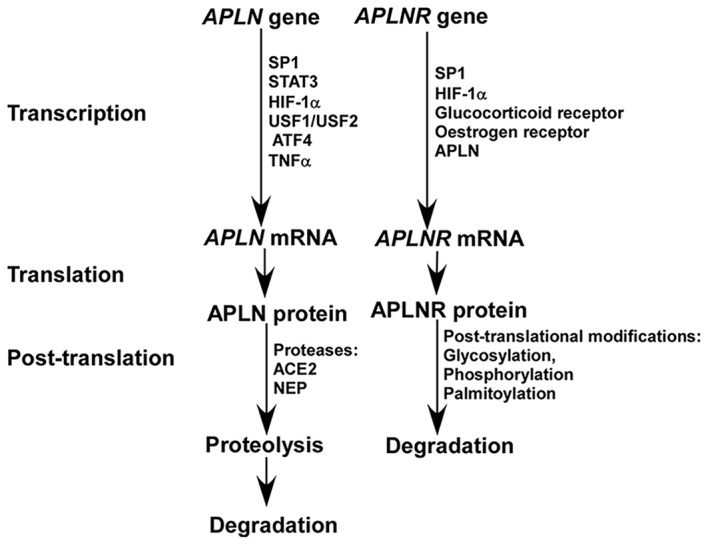
**The expression of apelin and apelin receptor (APLNR) is differentially regulated at transcriptional and post-translational levels**.

Apelin is expressed in both brain and peripheral tissues in human. In the brain, apelin is expressed in thalamus, frontal cortex and hippocampus, while it is also expressed in placenta, heart, lung and other peripheral tissues. The expression of apelin is controlled at both transcriptional and post-translational levels (Figure 1). First, multiple transcriptional factors are involved in the transcriptional regulation of apelin, such as Sp1 transcription factor (SP1), signal transducer and activator of transcription 3 (STAT3), hypoxia inducible factor 1 alpha (HIF-1α), upstream transcription factor 1/upstream transcription factor 2 (USF1/USF2) and activating transcription factor 4 (ATF4; Wang et al., 2006; Han et al., 2008; Lv et al., 2013; Jeong et al., 2014; He et al., 2015). Moreover, tumor necrosis factor alpha (TNF-α) increases the expression of APLN mRNA via phosphatidyl Inositol 3-kinase (PI3K) activation (Daviaud et al., 2006). In addition, apelin has a high turnover rate, with a half life of less than 8 min (Aydin et al., 2014; Juhl et al., 2016). Two identified proteases, angiotensin-converting enzyme 2 (ACE2) and metalloprotease (NEP), are involved in apelin proteolysis, which partially or fully inactivate the binding activity of apelin to its receptor and contribute to its degradation (Kalea and Batlle, 2010; McKinnie et al., 2016; Wang W. et al., 2016).

### APLNR

APLNR is the unique receptor of apelin, while apelin has until recently been considered the only endogenous ligand of APLNR. Recently, Chng et al. (2013) reported that elabela, also named as apela or toddler, is another endogenous ligand of APLNR in zebrafish, which is essential for early cardiovascular development. However, the expression of elabela is restricted to pluripotent cells and kidney in human (Wang et al., 2015).

The APLNR gene is located on chromosome 11q12, spanning 3877bp genomic DNA without introns. Human APLNR, consisting of 380 amino acids, shares more than 90% amino acid sequence homology to mouse and rat APLNR. It is widely distributed in human tissues. In human brain, APLNR mRNA is highly expressed in caudate nucleus, corpus callosum and hippocampus (Matsumoto et al., 1996; Edinger et al., 1998; Medhurst et al., 2003). Recently, its expression is also detected in the cortex (Hansen et al., 2007). The aforementioned evidence suggests that APLNR has important functions in multiple brain regions, which has not been fully investigated. APLNR, a typical G protein coupled receptor (GPCR), mainly mediates signal transduction via Gα subunit (Gα_i_ or Gα_q_) of heterotrimeric G protein. For example, Gα_i_ contributes to PI3K/serine/threonine kinase (PI3K/AKT) activation and extracellular signal-regulated kinase (ERK) activation, while Gα_q_ contributes to phospholipase C beta-protein kinase C (PLCβ-PKC) activation (Chapman et al., 2014). In addition, it may mediate signal transduction through a β-arrestin-dependent mechanism (Chapman et al., 2014). However, the exact role of the β-arrestin-dependent pathway in signal transduction remains elusive.

A number of transcription factors are implicated in the regulation of APLNR transcription, as multiple functional binding sites are located in the promoter region of the APLNR gene, including SP1, HIF-1α, glucocorticoid receptor and estrogen receptor (O’Carroll et al., 2006; Figure 1). Insulin is also involved in the regulation of APLNR expression (Dray et al., 2010). In addition, apelin deficiency reduces the level of *APLNR* mRNA (Wang et al., 2009). Hypoxia promotes both apelin and APLNR expression accompanying with HIF-1α induction, suggesting that upregulation of APLNR may be mediated by both apelin and/or HIF-1α (He et al., 2015). Moreover, APLNR undergoes multiple post-translational modifications, including glycosylation, phosphorylation and palmitoylation, which may contribute to the alteration of APLNR at the protein level by affecting its turnover rate (Figure 1; O’Carroll et al., 2013; Chen et al., 2014). For example, the phosphorylation status of regulator of calcineurin 1(RCAN1) has a significant effect on its degradation rate (Genescà et al., 2003).

## Temporal Expression of Apelin/APLNR in Ischemic Stroke

### Increased Expression of Apelin/APLNR During the Ischemic Phase of Ischemic Stroke

**Figure 2:**
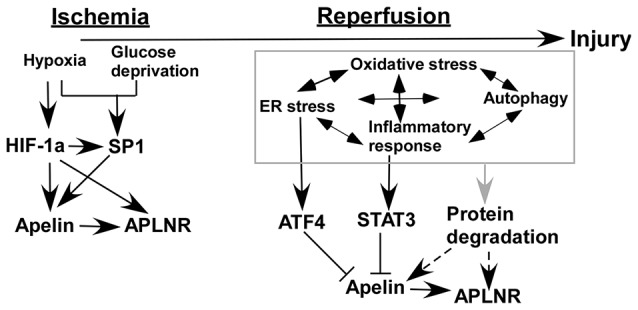
**Temporal expression of apelin/APLNR in ischemic stroke.** HIF-1α and SP1 transcription factor (SP1) contribute to the upregulation of apelin/APLNR during the ischemic phase, while multiple mechanisms are involved in the reduction of apelin/APLNR during the reperfusion phase.

Ischemia/reperfusion injury occurs in both heart and brain, accompanied by many common pathophysiological alterations. Numerous* in vitro* and* in vivo* studies showed that the expression of apelin and APLNR is altered by ischemia/reperfusion injury via a number of mechanisms (Figure 2). First, hypoxia and glucose deprivation, the major consequences of ischemia, are implicated in the aberrant expression of apelin and APLNR. The expression of apelin and APLNR is increased under hypoxic conditions, which is mediated by the induction of HIF-1α (He et al., 2015). HIF-1α can functionally bind to hypoxia response elements (HREs) within the promoter region of the apelin and APLNR genes, leading to the upregulation of apelin and APLNR (Ronkainen et al., 2007; He et al., 2015). In addition, glucose deprivation upregulates apelin and APLNR expression at early stages (Zhang et al., 2009). Moreover, the increase of SP1 promotes apelin and APLNR expression in ischemic neurons at the early stage, which may be mediated by HIF-1α induction (Woo et al., 2012; O’Carroll et al., 2006; Yeh et al., 2011; Lv et al., 2013). Consistently, the expression of both apelin and APLNR is increased by ischemia in rat hearts (Atluri et al., 2007; Sheikh et al., 2008).

### Reduced Expression of Apelin/APLNR During the Reperfusion Phase of Ischemic Stroke

Recent studies indicate that the expression of apelin and APLNR may be temporally regulated in ischemic stroke. The expression of apelin and APLNR is only increased early after ischemia in heart (Rastaldo et al., 2011). Although glucose deprivation upregulates the expression of apelin and APLNR at early stages, it significantly reduces apelin and APLNR expression at later stages (Zhang et al., 2009). The later-stage reduction of apelin and APLNR may be associated with a plethora of mechanisms in ischemic stroke (Figure 2). For example, ER stress is a major mechanism contributing to ischemia/reperfusion injury in both heart and brain (Morimoto et al., 2007; Nakka et al., 2010; Tao et al., 2011; Xin et al., 2014). However, it is only activated at the reperfusion stage but not at the ischemic stage in both heart and brain (Morimoto et al., 2007; Nakka et al., 2010; Tao et al., 2011). ATF4, an important mediator of ER stress, significantly reduces apelin expression via a p38 mitogen-activated protein kinase (p38-MAPK) dependent pathway (Jeong et al., 2014), suggesting that ER stress may contribute to the reduction of apelin expression during the reperfusion phase (Figure 2). Subsequently, APLNR may be downregulated by apelin reduction (Wang et al., 2009). In addition, inflammatory responses may be implicated in the regulation of apelin and APLNR expression mediated by STAT3, as it directly regulates the promoter activity of the apelin gene (Han et al., 2008; Liang et al., 2016). Interestingly, STAT3 is only activated during the reperfusion phase but not the ischemic phase. The inhibition of STAT3 signaling dramatically improves the outcome of ischemic stroke, suggesting that STAT3 may contribute to the reduction of apelin expression during the reperfusion phase (Figure 2; Li and Zhang, 2003; Yu et al., 2013). Moreover, the interplay among oxidative stress, ER stress, autophagy and inflammatory responses may be implicated in the temporal expression of apelin and APLNR (Sheng et al., 2012; Mei et al., 2015).

Understanding the temporal expression of apelin/APLNR is critical for defining the timing of treatment with apelin or APLNR agonists. For example, Rastaldo et al. (2011) reported that apelin can only reduce infarct size of heart when it is administrated at the reperfusion phase. Therefore, the temporal expression of apelin/APLNR in ischemic stroke and the underlying mechanisms need to be further investigated, which is crucial for the therapeutic application of apelin or APLNR agonists to treat ischemic stroke.

## Protective Effects of Apelin/APLNR in Ischemic Stroke and the Underlying Mechanisms

### Apelin/APLNR Inhibits Cell Death in Ischemic Stroke

**Figure 3:**
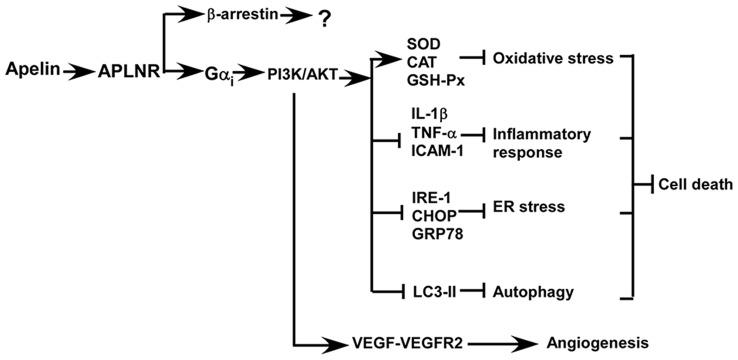
**Apelin/APLNR improves the recovery from ischemic stroke by inhibiting cell death and facilitating angiogenesis via Gα_i_- dependent mechanisms, while the role of β–arrestin-dependent pathways in ischemic stroke is unknown**.

Accumulated evidence indicates that apelin/APLNR improves the recovery from ischemic stroke by inhibiting cell death and facilitating angiogenesis, which are mediated by the activation of PI3K/AKT and ERKs signaling pathways, respectively (Figure 3; Chuang et al., 2011; Tao et al., 2011; Gu et al., 2013; Wang et al., 2013; Zhu et al., 2013; Yang et al., 2014; Chen et al., 2015; Liu et al., 2015; Huang et al., 2016; Novakova et al., 2016; Zou et al., 2016). Oxidative stress is a major mechanism of ischemia/reperfusion injury, leading to cell death. Mitochondria-derived oxidative stress is involved in ischemia/reperfusion injury by causing an overload in reactive oxygen species (ROS) and the increased ROS further promotes mitochondria impairment, forming a vicious feedback loop. Blocking this vicious cycle by suppressing oxidative stress is a major mechanism of apelin/APLNR’s protective effects on ischemic stroke (Figure 3). First, apelin reduces ischemia/reperfusion injury-induced oxidative stress by increasing the activity of antioxidant enzymes in kidneys, such as superoxide dismutase (SOD), catalase (CAT) and glutathione peroxidase (GSH-Px) (Bircan et al., 2016). Than et al. (2014) showed that apelin promotes the expression of antioxidant enzymes via MAPK kinase/ERK pathway in adipocytes. Moreover, apelin analogs inhibit mitochondrial ROS generation in hearts (Pisarenko et al., 2015). The aforementioned evidence suggests that the protective effect apelin/APLNR on oxidative stress-induced cell death may be mediated by suppressing ROS generation and promoting ROS scavenging, which remains to be investigated.

Oxidative stress not only promotes cell death but also contributes to the induction of inflammatory responses, which is another mechanism contributing to cell death in ischemic stroke. Apelin inhibits inflammatory response via reducing the generation of inflammatory cytokines (Figure 3). For example, apelin significantly reduces the expression of inflammatory cytokines in rat brains, such as interleukin 1 beta (IL-1β), TNF-α and intercellular adhesion molecule 1(ICAM-1), and inhibits cell apoptosis in rats with ischemic stroke (Chen et al., 2015; Xin et al., 2015; Yan et al., 2015). Moreover, apelin markedly reduces microglial activation and their recruitment to the ischemic penumbra, which is associated with a decrease in cell death (Chen et al., 2015).

Apelin can protect cells from apoptosis by preventing ER stress, which is activated by ischemia/reperfusion injury in both brain and heart (Figure 3; Morimoto et al., 2007; Nakka et al., 2010; Tao et al., 2011; Xin et al., 2014). ER stress is only activated at the reperfusion stage but not activated at the ischemic stage, suggesting that administration of apelin during the reperfusion stage may be effective in protecting cells from ER stress-induced apoptosis (Morimoto et al., 2007; Nakka et al., 2010; Tao et al., 2011). For example, Nakka et al. (2010) showed that ER stress is activated around 3–6 h after reperfusion in rat brains. Consistently, Tao et al. (2011) showed that ER stress is activated 2 h after reperfusion in heart. Moreover, apelin significantly reduces ER stress and infarct size via a PI3K/AKT-dependent pathway (Tao et al., 2011). Importantly, apelin has a long-term effect on the inhibition of ER stress, beyond 24 h after reperfusion (Tao et al., 2011). However, the expression of apelin is downregulated by ER stress (Jeong et al., 2014). The aforementioned evidence indicates that apelin reduction and the activation of ER stress may form a vicious feedback loop during the reperfusion phase, similar to mitochondria-induced stress, which potentiates ER stress-induced apoptosis. Thus, apelin administration may block this vicious cycle to inhibit ER stress-induced apoptosis in ischemic stroke.

Growing evidence indicates that the protective effect of apelin on ischemia/reperfusion-induced apoptosis may be associated with its role in the regulation of autophagy (Figure 3). First, apelin is able to inhibit glucose deprivation-induced autophagy, contributing to the protective effect on cardiomyocytes. This effect is associated with the activation of PI3K/AKT/mechanistic target of rapamycin (mTOR) pathway (Jiao et al., 2013). In addition, apelin inhibits hypoxia-induced autophagy via activating PI3K/AKT/mTOR pathway in muscle cells (Zhang et al., 2014a). Moreover, apelin attenuates traumatic brain injury-induced damage by suppressing autophagy (Bao et al., 2015).

### Apelin/APLNR Promotes Angiogenesis in Ischemic Stroke

Post-stroke angiogenesis has a beneficial effect on cell survival and stroke recovery. Apelin not only protects cells from death but also promotes angiogenesis against ischemic stroke and improves stroke recovery (Chen et al., 2015; Huang et al., 2016). Apelin facilitates angiogenesis and blood flow restoration, which is dependent on an increase in vascular endothelial growth factor- vascular endothelial growth factor receptor 2 (VEGF-VEGFR2) signaling*.* In addition, PI3K/AKT and ERKs signaling pathways play a crucial role in apelin/APLNR-induced angiogenesis in ischemic stroke, which may be mediated by VEGF-VEGFR2 (Wang et al., 2013; Chen et al., 2015; Liu et al., 2015; Novakova et al., 2016). Consistently, loss of apelin impairs the angiogenesis and functional recovery (Wang et al., 2013).

## Conclusions and Perspectives

The aforementioned evidence suggests that activating apelin/APLNR system may be a potent approach for protecting against ischemic stroke and improving recovery following an ischemic event. However, several critical issues need to be well resolved. First, the temporal expression of apelin/ APLNR during ischemic stroke needs to be determined, as it is crucial for deciding the timing of treatment with apelin or APLNR agonists. Based on the alteration of apelin/APLNR expression in ischemia/reperfusion injury of heart, application of apelin after thrombolytic therapy may be an effective approach to protect penumbra cells from apoptosis. Moreover, modified apelin or APLNR agonists with longer half life need to be developed, as the half life of apelin is too short to be applied clinically. Finally, non-invasive drug delivery methods need to be further explored. As APLNR is widely expressed in peripheral tissues, systematic delivery may generate more side effects. Thus, apelin was administered through lateral ventricle injection in most* in vivo* studies. However, this approach is not suitable for clinical application. A recent study has applied intranasal delivery of apelin for stroke treatment, shedding a light on exploiting non-invasive apelin application to treat ischemic stroke (Chen et al., 2015).

## Author Contributions

YW formulated the study, wrote the manuscript and designed the figures. XW, XZ, BC and GL wrote the manuscript. BB provided intellectual thoughts, revised the manuscript and was the project leader.

## Funding

This work was supported by grants from the National Natural Science Foundation of China (81070961 to BB, 81571334 to GL, 81671276 to BC), the Jining science and technology development plan (2013jnwk75 to BB), Natural Science Foundation of Shandong Province (ZR2016HM30 to YW, ZR2011HM023 to GL, ZR2014HL040 to BC, ZR2010HL057 and ZR2012HL24 to BC), Science and Technology Project of Higher education of Shandong Province (J10LF01 to GL), the Development of Medical Science and Technology Project of Shandong Province (2011HZ011 to GL), Postgraduate Education Innovation Program of Shandong Province (SDYY15012 to GL) and Research Project of Teaching Reform in Undergraduate Colleges and Universities in Shandong Province (2015M049 to GL), Project of Shandong Province Higher Educational Science and Technology Program (J12LE10 to BC).

## Conflict of Interest Statement

The authors declare that the research was conducted in the absence of any commercial or financial relationships that could be construed as a potential conflict of interest.

## Abbreviations

ACE2: angiotensin-converting enzyme 2
AKT: serine/threonine kinase
APLN: apelin
APLNR: apelin receptor
ATF4: activating transcription factor 4
CAT: catalase
ER: endoplasmic reticulum
ERK: extracellular signal-regulated kinase
GSH-Px: glutathione peroxidase
HIF-1α: hypoxia inducible factor 1 alpha
HREs: hypoxia response elements
ICAM-1: intercellular adhesion molecule 1
IL-1β: interleukin 1 beta
mTOR: mechanistic target of rapamycin
NEP: metalloprotease
p38-MAPK: p38 mitogen-activated protein kinase
PCSK3: proprotein convertase subtilisin/kexin 3
PI3K: phosphatidyl Inositol 3-kinase
PLCβ-PKC: phospholipase C beta-protein kinase C
ROS: reactive oxygen species
SOD: superoxide dismutase
SP1: Sp1 transcription factor
STAT3: signal transducer and activator of transcription 3
TNF-α: tumor necrosis factor alpha
USF1/USF2: upstream transcription factor 1/upstream transcription factor 2
VEGF: vascular endothelial growth factor
VEGFR2: vascular endothelial growth factor receptor 2.
